# Supplementary material for: Hypomethylating agents increase L1 retroelement expression without inducing novel insertions in myeloid malignancies
Source: Mol Oncol. 2025 Sep 4;19(10):2764–75. doi: 10.1002/1878-0261.70111 (PMC12515712; doi:10.1002/1878-0261.70111)
Supplement: Supplementary file 1 — Fig. S1. Strategy for gating positive cells using an isotype control for ORF1p staining and secondary antibody only for ORF2p staining in flow cytometry experiments. Fig. S2. Confocal microscopy detection of ORF1p in MCF‐7 cell line used as a positive control. Fig. S3. HEK293T cell transfection with ORF1 and ORF2 constructs. Fig. S4. The quantification of ORF1p subcellular localization in DAMI cells treated with Aza‐dC. Fig. S5. PCR validation of candidate L1 insertions detected by NGS. [file MOL2-19-2764-s002.docx]

**Figure S1**


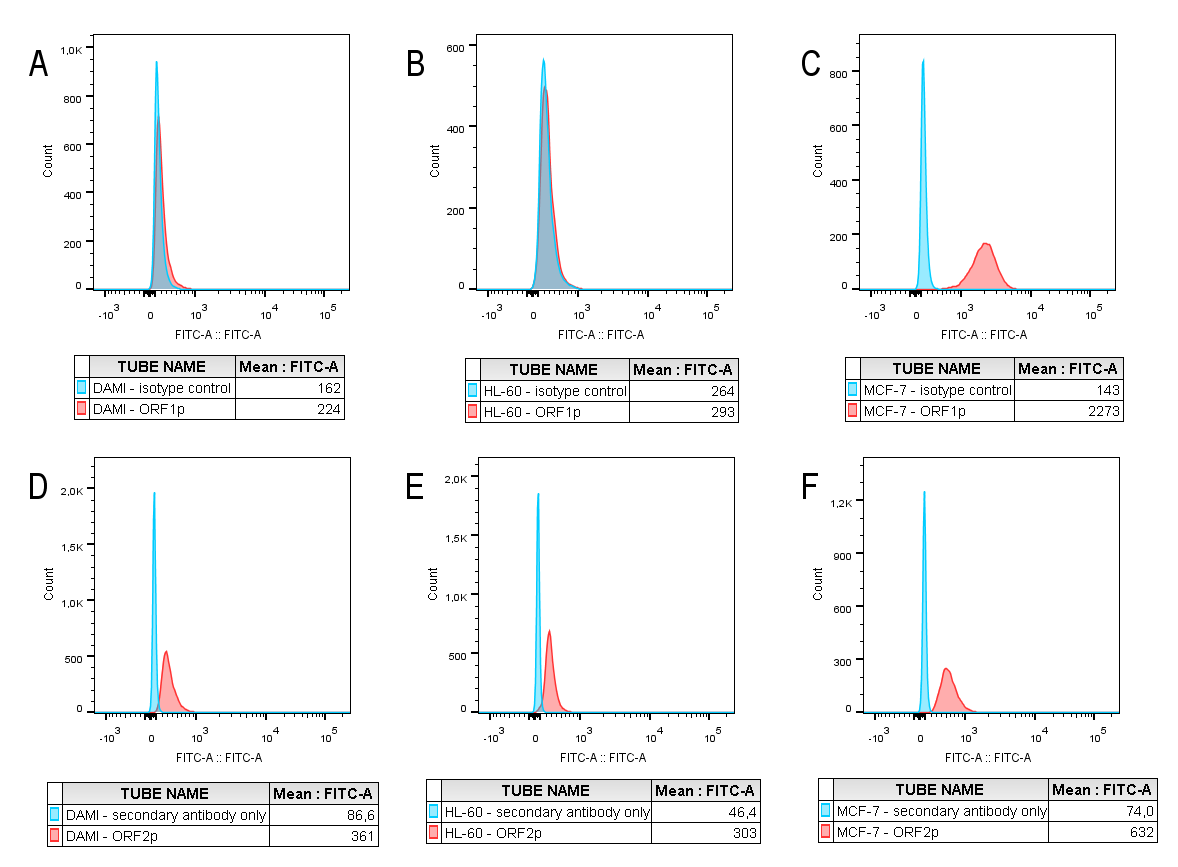


**Figure S1**. **Strategy for gating positive cells using an isotype control for ORF1p staining and secondary antibody only for ORF2p staining in flow cytometry experiments:** Controls used for background signal subtraction in ORF1p and ORF2p flow cytometry analysis. Histograms show fluorescence intensity of untreated (A, D) DAMI, (B, E) HL-60, and (C, F) MCF-7 cell lines. For ORF1p analysis (A–C), each cell line's mean fluorescence intensity (MFI) was subtracted by the MFI of its respective isotype control. For ORF2p analysis (D–F), MFI values were subtracted by the MFI of the sample stained with secondary antibody only, as no suitable isotype control with a lower signal than the ORF2p antibody was available (two isotype controls from two manufacturers were tested: Rockland, 003-012; Abcam, ab37382). MFI values for each condition are provided in the tables below each histogram.

**Figure S2**


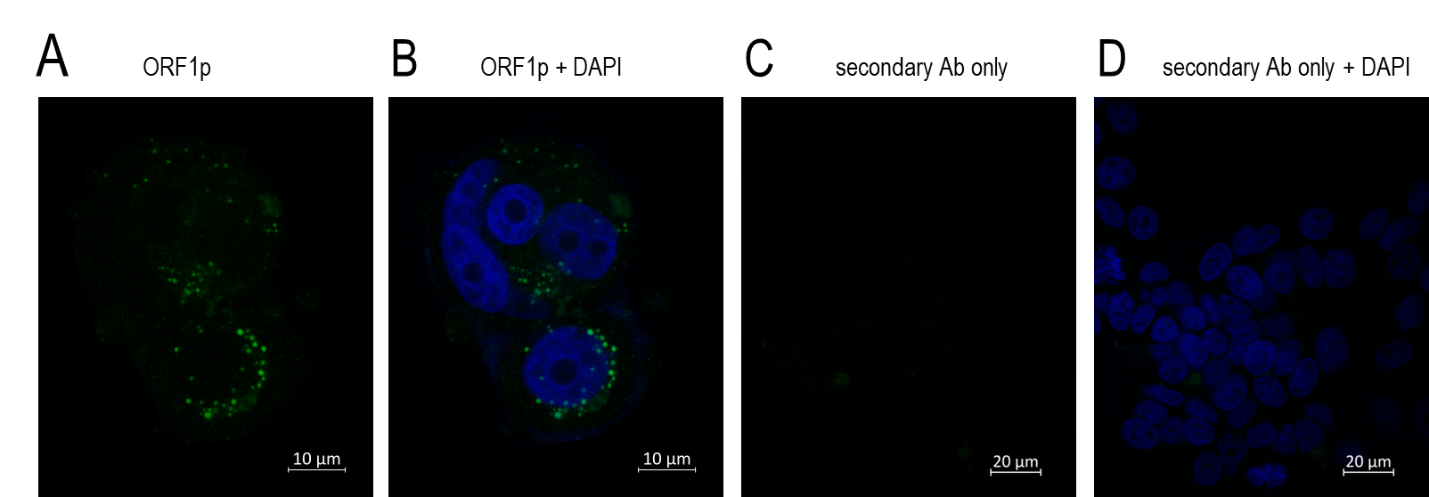


**Figure S2. Confocal microscopy detection of ORF1p in MCF-7 cell line used as a positive control**: (A) The immunofluorescence signal of ORF1p (green). (B) The immunofluorescence signal of ORF1p (green) combined with the nuclei signal (DAPI, blue). Negative control: Cells incubated with the secondary antibody only, displayed in the green channel (C) and in the merged green and blue channels (D). Scale bars represent 10 μm (A, B) or 20 μm (C, D).

**Figure S3**


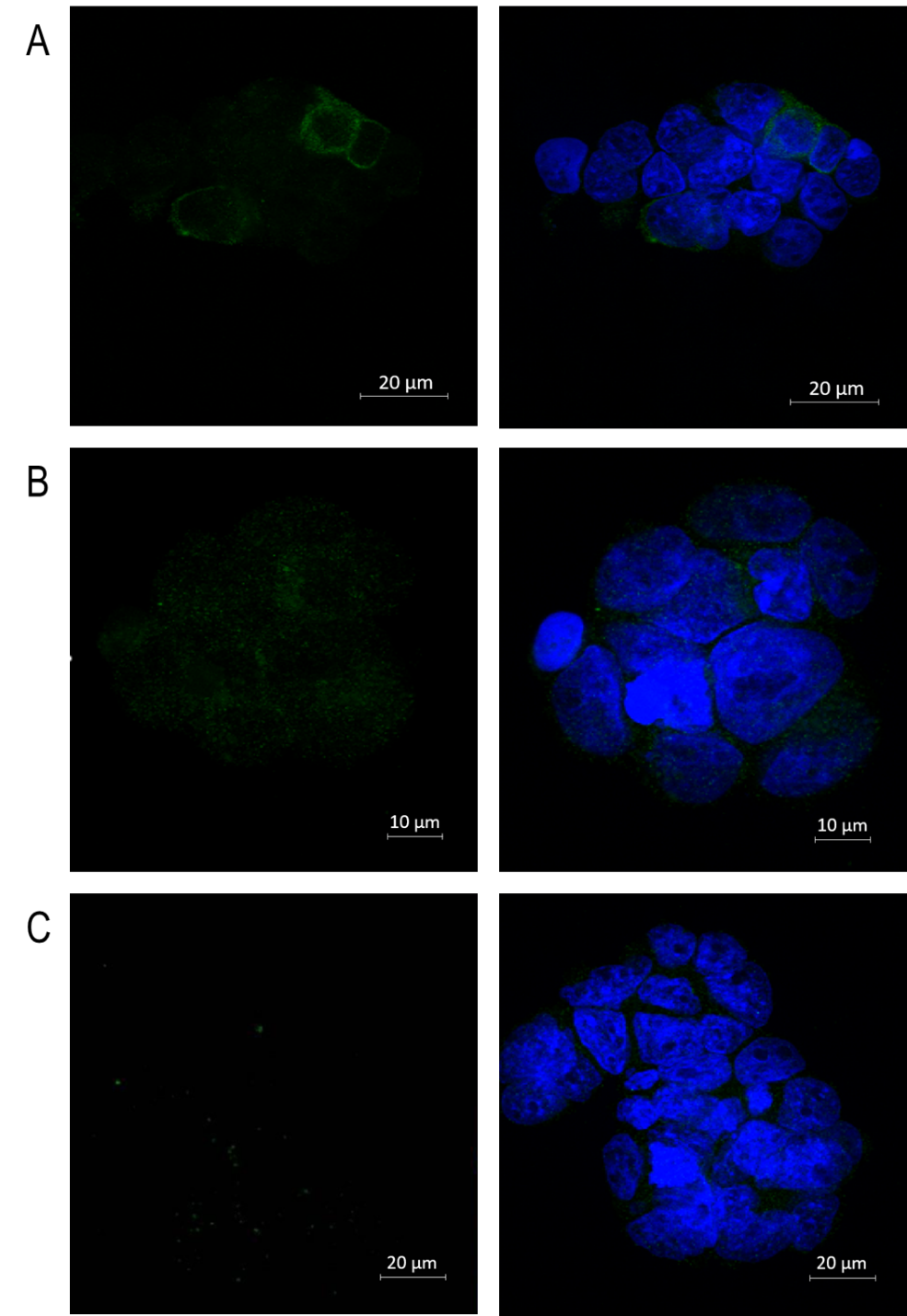


**Figure S3. HEK293T cell transfection with ORF1 and ORF2 constructs:** The cells were transfected with pBudORF1-CH (A), pBudORF2-CH (B) (Addgene), and non-transfected control (C). Left panel: The immunofluorescence signal of ORF1p/ORF2p (green) in HEK293T cells. Right panel: The immunofluorescence signal of ORF1p/ORF2p (green) combined with the nuclei signal (DAPI, blue). Scale bars represent 10 μm (B) or 20 μm (A, C).

**Figure S4**


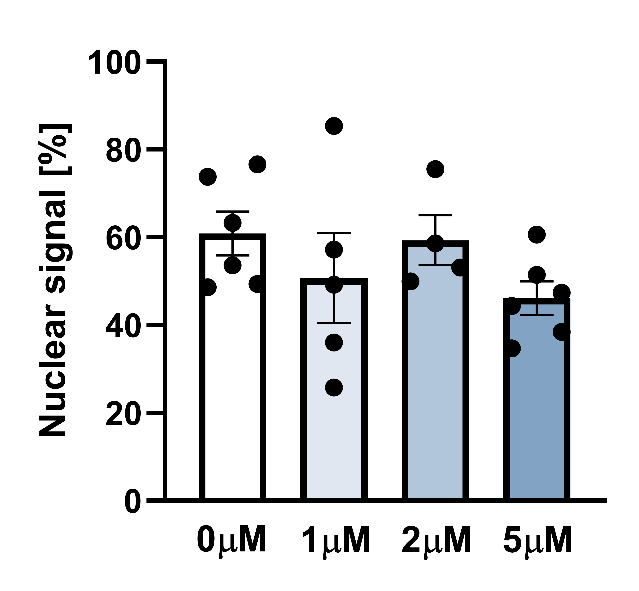


**Figure S4**. **The quantification of ORF1p subcellular localization in DAMI cells treated with Aza-dC**: The bar graph depicts the percentage of nuclear ORF1p signal in DAMI cells treated with Aza-dC (0, 1, 2, and 5 μM). The quantification was performed in confocal microscopy images (N=21), with the number of analyzed images as follows: 0 μM (N=6), 1 μM (N=5), 2 μM (N=4), and 5 μM (N=6). Data are presented as mean ± standard error of the mean. Each dot represents an individual image. No consistent trend was observed across different Aza-dC concentrations.

**Figure S5**


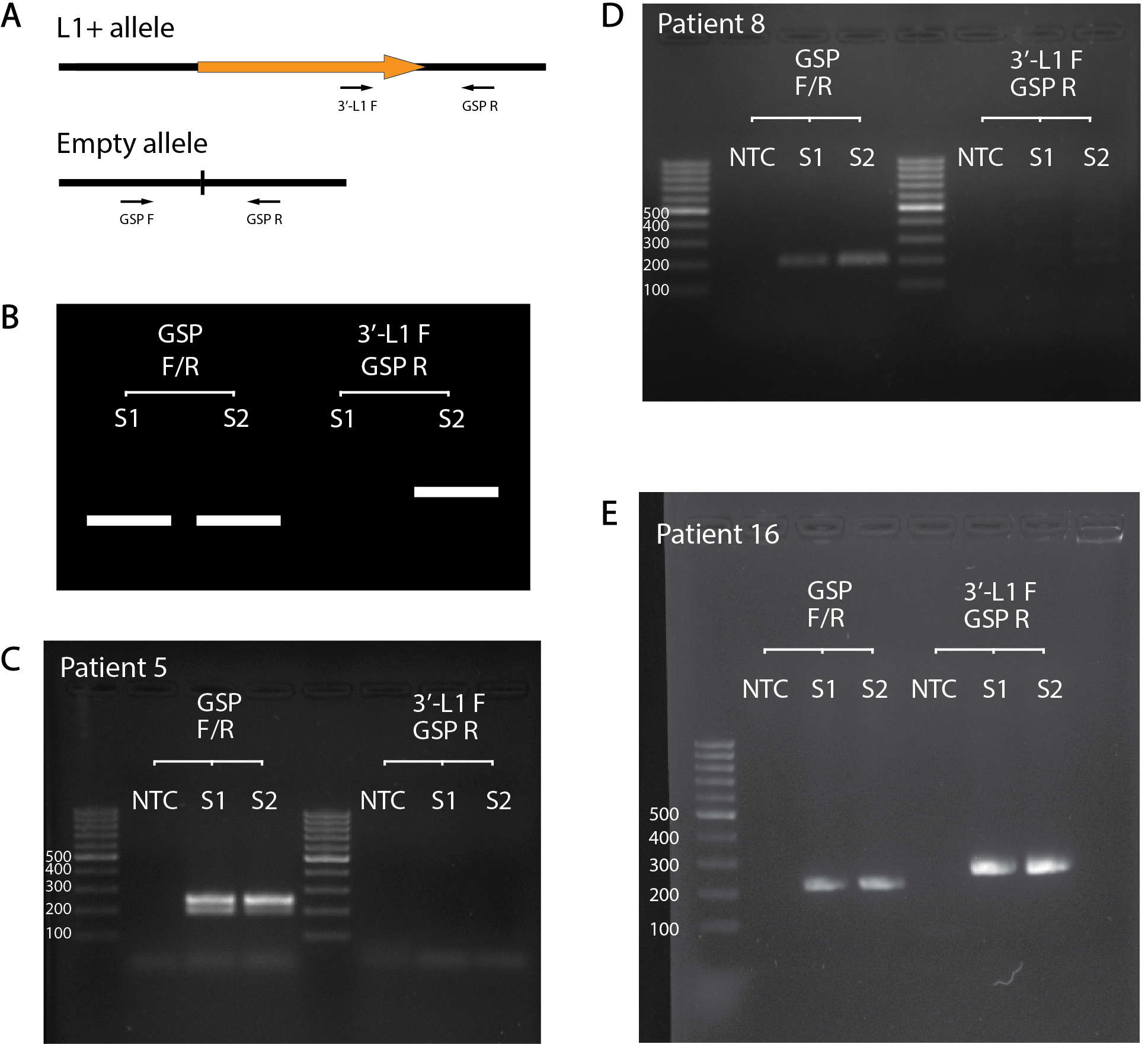


**Figure S5. PCR validation of candidate L1 insertions detected by NGS.** The approach is based on locus-specific PCR with primers designed for every candidate insertion. (A) We used a universal forward primer (3’-L1 F) specific for the 3’-end of every L1HS insertion (orange arrow) in combination with the genomic locus-specific reverse primer (GSP R) to capture the allele carrying novel insertion. In parallel, genomic locus-specific forward and reverse primers (GSP F and GSP R) were used to amplify an empty allele. Both PCR reactions were carried out for all consecutive samples of a patient or cell-line culturing condition. PCR products were then analyzed using agarose gel electrophoresis to reveal novel L1 insertions; a hypothetic positive result is illustrated (B), showing novel insertion in the follow-up sample S2, which was not present in the baseline sample S1. The results for the three MDS patients (5, 8, 16) with candidate insertions in the samples taken during Aza therapy turned out negative (C-E). Patients 5 and 8 had false positive results by NGS in samples S2, while Patient 16 had false negative result in sample S1. Supplementary Tables S4 and S5 list PCR primers for all validation reactions. NTC: non-template control; S1: before Aza; S2: during Aza therapy.
